# Prevalence of Slow-Growth Vancomycin Nonsusceptibility in Methicillin-Resistant Staphylococcus aureus

**DOI:** 10.1128/AAC.00452-17

**Published:** 2017-10-24

**Authors:** Yuki Katayama, Takuya Azechi, Motoyasu Miyazaki, Tohru Takata, Miwa Sekine, Hidehito Matsui, Hideaki Hanaki, Koji Yahara, Hiroshi Sasano, Kota Asakura, Tomoiku Takaku, Tomonori Ochiai, Norio Komatsu, Henry F. Chambers

**Affiliations:** a Department of Microbiology, Faculty of Medicine, Juntendo University, Tokyo, Japan; b Department of Pharmacy, Juntendo University Hospital, Tokyo, Japan; c Department of Pharmacy, Fukuoka University Chikushi Hospital, Chikushino, Japan; d Department of Infection Control, Fukuoka University Hospital, Fukuoka, Japan; e Infection Control Research Center, Kitasato Institute for Life Science, Kitasato University, Tokyo, Japan; f Department of Bacteriology II, National Institute of Infectious Diseases, Tokyo, Japan; g Division of Hematology, Department of Internal Medicine, Juntendo University, Tokyo, Japan; h Department of Medicine, University of California San Francisco, San Francisco, California, USA

**Keywords:** HA-MRSA, MRSA, hVISA, mupirocin, ppGpp, recurrent infection, slow VISA, stringent response, tolerance, vancomycin resistance

## Abstract

We previously reported a novel phenotype of vancomycin-intermediate Staphylococcus aureus (VISA), i.e., “slow VISA,” whose colonies appear only after 72 h of incubation. Slow-VISA strains can be difficult to detect because prolonged incubation is required and the phenotype is unstable. To develop a method for detection of slow-VISA isolates, we studied 23 slow-VISA isolates derived from the heterogeneous VISA (hVISA) clinical strain Mu3. We identified single nucleotide polymorphisms (SNPs) in genes involved in various pathways which have been implicated in the stringent response, such as purine/pyrimidine synthesis, cell metabolism, and cell wall peptidoglycan synthesis. We found that mupirocin, which also induces the stringent response, caused stable expression of vancomycin resistance. On the basis of these results, we developed a method for detection of slow-VISA strains by use of 0.032 μg/ml mupirocin (Yuki Katayama, 7 March 2017, patent application PCT/JP2017/008975). Using this method, we detected 53 (15.6%) slow-VISA isolates among clinical methicillin-resistant S. aureus (MRSA) isolates. In contrast, the VISA phenotype was detected in fewer than 1% of isolates. Deep-sequencing analysis showed that slow-VISA clones are present in small numbers among hVISA isolates and proliferate in the presence of vancomycin. This slow-VISA subpopulation may account in part for the recurrence and persistence of MRSA infection.

## INTRODUCTION

Two major issues with vancomycin as a treatment for severe methicillin-resistant Staphylococcus aureus (MRSA) infections, including bacteremia, are treatment failure (1–3) and recurrent infection. The role of bacterial factors in poor outcomes has not been well defined. To address these issues, we have been focusing on the possible involvement of a novel phenotype, namely, slow-growing vancomycin-intermediate S. aureus (“slow VISA”). Slow-VISA strains have the following three major growth properties: (i) a low growth rate, (ii) resistance to concentrations of vancomycin higher than those resisted by other extant clinical strains of VISA (MIC ≥ 8 μg/ml), and (iii) unstable resistance and colony morphology. *rpoB* or *rpoC* mutations can produce the slow-VISA phenotype (4, 5).

Detection of slow-VISA and heterogeneous VISA (hVISA) strains (6) is problematic. The vancomycin MICs of hVISA are, by definition, less than 4 μg/ml (the susceptibility breakpoint for VISA), and thus hVISA is classified as vancomycin-susceptible S. aureus (VSSA) by MIC testing. Nonetheless, hVISA is distinguishable from VSSA by the presence of cells resistant to 4 μg/ml vancomycin within the cell population, at a frequency of 1 × 10^−6^ or greater. hVISA is thought to be an intermediate step in the development of resistance from VSSA to VISA. hVISA and slow-VISA cells may also lose their resistance in the process of repeated isolation and incubation or may not be detected at all under the standard incubation period of 48 h recommended by the Clinical and Laboratory Standards Institute (CLSI) guidelines.

In this study, we found numerous mutations in hVISA strain Mu3-derived slow-VISA strains, in genes involved in various metabolic pathways, including purine/pyrimidine synthesis and 5P-d-ribosyl-1-pyrophosphate (PRPP) synthesis, as well as in *rpoB* and *rpoC*. These genes are known to be involved in the stringent response, a phenomenon originally identified in cells subjected to the stress of amino acid starvation, during which cells undergo massive physiological changes to save energy and conserve cellular resources (7). Mupirocin also induces the stringent stress response and amino acid starvation by blocking the charging of isoleucyl-tRNA with isoleucine through competition with Ile-AMP for isoleucyl-tRNA synthetases. Accumulation of unloaded isoleucyl-tRNA results in upregulation of the isoleucyl-tRNA synthetase (*ileS*) gene (8–10). Mupirocin-mediated amino acid starvation in S. aureus results in downregulation of genes involved in nucleotide biosynthesis, DNA metabolism, energy metabolism, and translation (11). The stringent response is mediated by (p)ppGpp, which regulates transcription by RNA polymerase (12). The stringent stress response induces high-level, homogeneous resistance to beta-lactams and depends on the transglycosylase activity of PBP2 (13, 14).

On the basis of these observations, we hypothesized that the mupirocin-induced stringent stress response could enhance expression of the slow-VISA phenotype. We developed a method for detecting slow-VISA strains by use of mupirocin and then investigated the prevalence of slow-VISA strains among clinical isolates by using the newly developed method.

## RESULTS

### Mutations present in slow-VISA mutants of Mu3.

**FIG 1:**
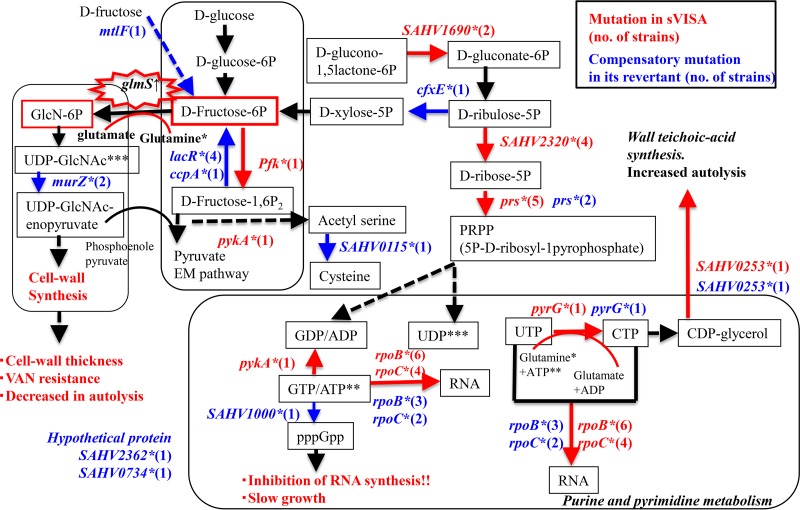
Proposed model for the mechanism underlying vancomycin resistance in slow-VISA (sVISA) strains. We compared genotypes between the 23 slow-VISA clinical isolates and 23 revertant hVISA strains shown in Table S3 in the supplemental material. Mutations in slow-VISA isolates and revertant hVISA strains are highlighted in red and blue, respectively.

We identified genetic mutations other than those in the *rpoB* and *rpoC* genes in 23 slow-VISA isolates, derived from the hVISA strain Mu3 in the presence of 6 μg/ml vancomycin for 72 to 144 h (4), and in their revertants. Mutations were detected in several genes involved in various metabolic pathways, such as cell envelope synthesis (*capP*), purine/pyrimidine synthesis (*pyrG* [CTP synthase], *pykA* [pyruvate kinase], and *SAHV1000* [GTP pyrophosphokinase]), the stringent response (PRPP synthesis, *prs* [ribose-phosphate pyrophosphokinase], *SAHV2320* [ribose 5-phosphate isomerase A], *cfxE* [ribulose phosphate 3-epimerase], and *SAHV1690* [similar to bifunctional oligoribonuclease] genes), and the Embden-Meyerhof (EM) pathway (*pfk*) (see Table S3 in the supplemental material). The *pykA* and GTP pyrophosphokinase genes have been associated with the VISA phenotype (5, 15). We mapped the main genetic mutations identified in the 23 slow-VISA isolates (Fig. 1, highlighted in red) and their 23 revertants (hVISA) (Fig. 1, highlighted in blue) to the corresponding metabolic pathways.

The vancomycin MIC was decreased compared to that of the mutant for 21 revertants obtained from the slow-VISA isolates (Table S3). Some mutations in the large-colony (LC) revertants occurred in the same genes but at different locations from those in the slow-VISA isolates (*rpoB*, *rpoC*, *prs*, *pyrG*, and *SAHV0253*). Mutations in other genes, for example, the *murZ* (encoding UDP-*N*-acetylglucosamine 1-carboxyvinyltransferase, the first enzyme in the biosynthetic pathway for production of peptidoglycan monomer units from UDP-GlcNAc), *lacR*, *ccpA* (16), *SAHV1000*, and *rpoC* genes, were detected in 10 LC revertants (Fig. 1).

### *In vivo* metabolomics analysis.

To determine the effect on metabolomics induced by mupirocin in the slow-VISA strain Mu3-6R, carrying the RpoB(R512P) mutation, we performed a comparative analysis of strains expressing three different vancomycin-nonsusceptible phenotypes. There were clear phenotypic differences between hVISA strain Mu3 and slow-VISA strain Mu3-6R, with each expressing different amounts of products related to the mutations shown in Fig. 1 (Table 1; Table S4). The concentrations of fructose 1,6-P_2_, fructose 6-P, ribose-5P, ribulose-5P, and PRPP were significantly decreased in slow-VISA strain Mu3-6R compared to those in hVISA strain Mu3 and VISA strain Mu3-5-5d in the absence and presence of mupirocin, as expected based on the mutations shown in Fig. 1.

**TABLE 1:** *In vivo* concentrations of major substances and ppGpp

Strain	Phenotype	Treatment	Concn (pmol/ml)^*a*^												
			Glucose	Fructose 1,6-P_2_	Fructose 6-P	Ribose 5-P	Ribulose 5-P	PRPP	GTP	GDP	ATP	UTP	UDP	CTP	ppGpp^*c*^
Mu3	hVISA	Drug-free	31,657	2,081	44	54	293	1,024	595	229	1,371	441	157	194	ND
		Mupirocin^*b*^	32,824	560	23	13	328	739	414	154	3,205	1,498	292	1,454	41 ± 6
Mu3-3-5d^*d*^	VISA	Drug-free	36,738	539	25	11	88	615	40	118	315	124	44	159	ND
		Mupirocin^*b*^	31,140	463	22	10	91	729	44	102	1,004	817	373	309	ND
Mu3-6R^*e*^	Slow VISA	Drug-free	31,384	472	12	13	149	504	245	31	1,415	1,717	498	534	ND
		Mupirocin^*b*^	30,759	231	11	7	87	283	175	56	2,566	1,364	235	1,362	12 ± 6

a Data calculated from two biological replicates. Each value indicates the average. Glucose concentration was used as a control value.

b The strain was induced with mupirocin (0.032 μg/ml) for 30 min. We detected more than 1 nmol/mg of CDW.

c Data are means ± standard deviations. ND, not detected. ppGpp was measured in triplicate.

d Mu3-3-5d showing the VISA phenotype carried the TagH(D26E) mutation.

e Mu3-6R showing the slow-VISA phenotype carried the RpoB(R512P) mutation.

Nucleotides, especially CTP, UTP, and UDP, used in peptidoglycan synthesis, were concentrated >4- to 10-fold in slow-VISA strain Mu3-6R compared to those in hVISA strain Mu3 and VISA strain Mu3-5-5d (Table 1). Low concentrations of GTP were apparent in slow-VISA strain Mu3-6R and VISA strain Mu3-5-5d in the absence and presence of mupirocin. However, the decrease in GTP concentration in Mu3-5-5d carrying TagH(D26E) was not accompanied by induction of (p)ppGpp (undetectable concentration) (Table 1). Mupirocin induces a decrease in GTP concentration (11, 17) and elicits a response in S. aureus that is linked to the stringent response induced by (p)ppGpp (8, 18).

### Mupirocin-induced stable resistance to and tolerance of vancomycin in slow-VISA strain Mu3-6R.

**FIG 2:**
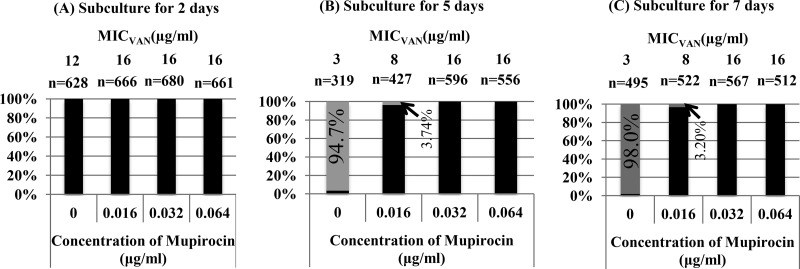
Stability of slow-VISA strain Mu3-6R showing high resistance to vancomycin induced by mupirocin. Black and gray bars indicate slow-VISA and hVISA strains, respectively; *n* is the number of tested colonies in a population. The *y* axis shows the proportion of hVISA cells among slow-VISA cells. The vancomycin MIC (MIC_VAN_) was determined after incubation for 48 h. Mu3-6R was subcultured without or with mupirocin at 0.016, 0.032, or 0.064 μg/ml for 2 days (A), 5 days (B), and 7 days (C).

To evaluate the effect of mupirocin on phenotypic expression of slow VISA, Mu3-6R was subcultured in an antibiotic-free medium for 2, 5, or 7 days. When the strain was subcultured for 5 days, more than 90% of the small colonies of slow-VISA strain Mu3-6R converted to the hVISA phenotype (vancomycin MIC = 3 μg/ml) (Fig. 2). The expression of the slow-VISA phenotype in Mu3-6R was maintained by cultivation in brain heart infusion (BHI) broth containing mupirocin at 0.032 or 0.064 μg/ml after subculture for 5 days (Fig. 2). The growth curve for Mu3-6R was shifted to the right (inhibition of growth) at a mupirocin concentration of 0.064 μg/ml (Fig. S1), and therefore a mupirocin concentration of 0.032 μg/ml was used for further experiments.

### Mupirocin accelerated the growth rate of Mu3-6R in the presence of vancomycin.

**FIG 3:**
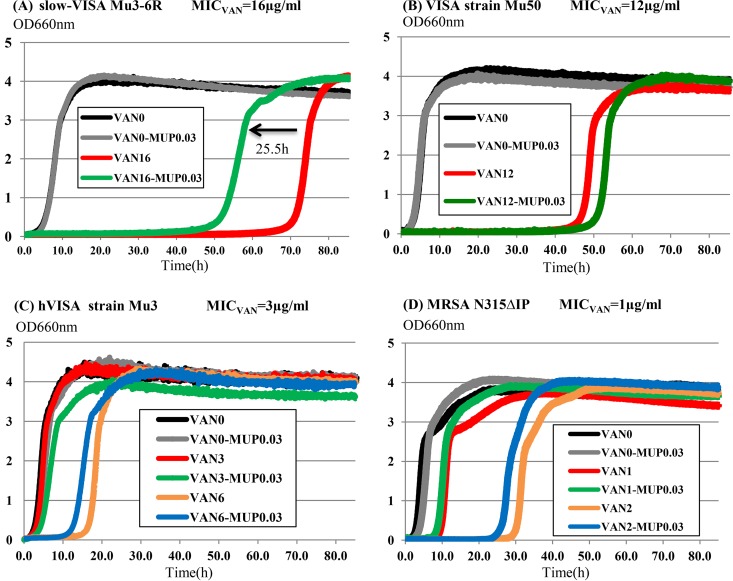
Increased growth rate of slow-VISA cells under the influence of mupirocin (0.032 μg/ml). Cultures were incubated in BHI broth. Black, drug-free conditions; gray, mupirocin; red, vancomycin at 1× MIC; green, vancomycin at 1× MIC plus mupirocin; orange, vancomycin at 2× MIC; blue, vancomycin at 2× MIC plus mupirocin. The growth curve was accelerated by 25.5 h compared to that for growth in BHI containing vancomycin at 16 μg/ml (1× MIC) without mupirocin. MIC_VAN_ indicates the vancomycin MIC for the strain.

When slow-VISA strain Mu3-6R was cultured in BHI broth containing 16 μg/ml of vancomycin (1× MIC) plus 0.032 μg/ml of mupirocin, the growth curve shifted to the left (i.e., growth was accelerated by 25.5 h) compared to that for growth in BHI broth without mupirocin (Fig. 3A). The growth curves for strains without the slow-VISA phenotype were only slightly affected by mupirocin (Fig. 3B to D).

### Reduced susceptibility of Mu3-6R to the bactericidal activity of vancomycin is induced by mupirocin.

**FIG 4:**
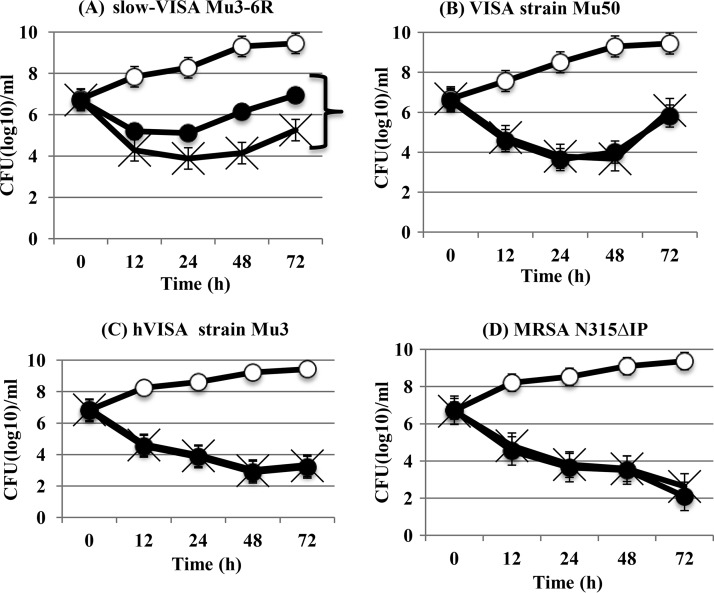
Mupirocin induced tolerance of vancomycin only in slow-VISA strain Mu3-6R. A time-kill assay was performed in BHI broth without a drug (○), with vancomycin at 20 μg/ml (×), and with vancomycin at 20 μg/ml plus mupirocin at 0.032 μg/ml (●) against slow-VISA strain Mu3-6R, VISA strain Mu50, hVISA strain Mu3, and VSSA (MRSA) strain N315ΔIP. Each symbol represents the average CFU (log_10_)/ml. The data are means ± standard deviations for triplicate wells.

Time-kill assays were performed to compare the *in vitro* activities of vancomycin at 20 μg/ml plus mupirocin at 0.032 μg/ml against slow-VISA, VISA, hVISA, and VSSA (MRSA) strains. Mupirocin induced tolerance (i.e., loss of bactericidal activity) of vancomycin in slow-VISA strains but not in the other strains (Fig. 4). The same effect on bactericidal activity was observed for vancomycin at 1× or 2× MIC (data not shown).

### Prevalence of clinical slow-VISA isolates among MRSA isolates from blood.

**FIG 5:**
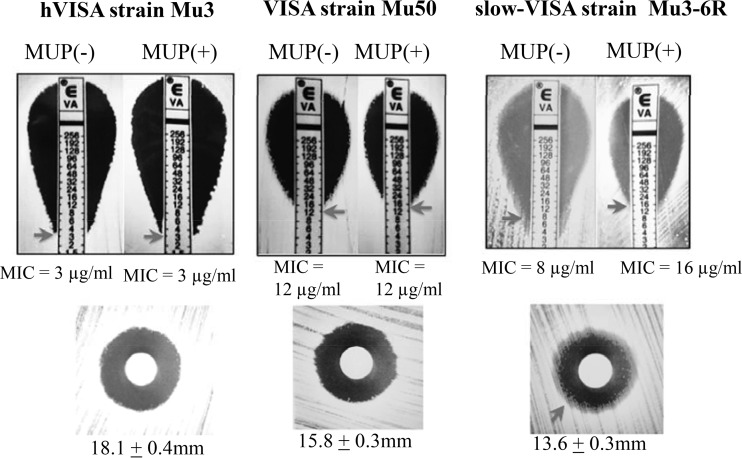
(Top) Etest in BHI agar plates containing mupirocin (MUP) at 0.032 μg/ml or no drug. After 48 h of incubation, the slow-VISA strain showed a stable MIC value. (Bottom) Vancomycin (30 μg/ml) disk diffusion test in BHI agar plates. Slow-VISA strains formed a hazy zone around the inhibition zone. The values given under each panel are the means and standard deviations for the inhibition zone diameter for 3 experiments.

We tested 340 Japanese clinical MRSA isolates (156 blood culture isolates from a single hospital [Table S1] and 184 randomly selected blood culture isolates collected from 9 hospitals [Table S2]) (19, 20) to determine the prevalence of the slow-VISA phenotype detected by use of mupirocin induction. Determination of the MIC on BHI agar containing mupirocin at 0.032 μg/ml detected the slow-VISA phenotype after 48 h of incubation instead of a 72-h or longer incubation in the absence of mupirocin (Table 2 and Fig. 5). In total, we identified 53 (15.6%) slow-VISA isolates (vancomycin MICs of >4 mg/liter). Less than 1% of the isolates were VISA strains (Table 2; Tables S1 and S2). The slow-VISA phenotype showed vancomycin MICs of >4 μg/ml after 48 h of incubation, with increasing resistance to vancomycin after 72 h of incubation. The hVISA strain Mu3 and the VISA strain Mu50 had stable vancomycin MICs of 3 μg/ml and 12 μg/ml over time, respectively, in the presence and absence of mupirocin (Table 2 and Fig. 5), and they showed no growth defects.

**TABLE 2:** Detection of slow-VISA cells among clinical MRSA isolates by use of mupirocin

Strain	Phenotype^*b*^		Vancomycin MIC (μg/ml) by Etest after various periods of incubation									
	CLSI classification (48 h)	New classification	Without antibiotics					With mupirocin at 0.032 μg/ml				
			48 h	72 h	96 h	120 h	144 h	48 h	72 h	96 h	120 h	144 h
209P	VSSA (MSSA)	VSSA (MSSA)	1.5	1.5	1.5	1.5	1.5	1.5	1.5	1.5	1.5	1.5
N315ΔIP	VSSA (MRSA)	VSSA (MRSA)	2	2	2	2	2	2	2	2	2	2
Mu3	hVISA	hVISA	3	3	3	3	3	3	3	3	3	3
Mu50	VISA	VISA	12	12	12	12	12	12	12	12	12	12
MI (HIP5827)	VISA	VISA	12	12	12	12	12	12	12	12	12	12
Mu3-6R	VISA	Slow VISA	8	8	12	12	16	16	16	16	16	16
Clinical blood culture isolates from 9 hospitals												
MRSA212	VSSA	VSSA	1.5	2	2	2	2	3	3	3	3	3
MRSA84	VSSA	hVISA	2	2	3	3	3	3	3	3	3	3
MRSA220	VSSA	hVISA	1.5	3	3	3	3	3	3	3	3	3
MRSA192	VSSA	hVISA	2	2	2	2	2	2	2	2	2	2
MRSA190	VISA	Slow VISA	4	8	8	8	8	12	12	12	12	12
MRSA194	VISA	Slow VISA	4	6	8	12	12	8	12	12	12	12
T series clinical blood culture isolates^*a*^												
T12	VISA	Slow VISA	4	4	8	8	8	8	8	8	8	8
T15	VISA	Slow VISA	4	6	8	8	8	8	8	8	8	8
T61	VISA	Slow VISA	4	6	8	8	8	8	8	8	8	8
T89	VISA	Slow VISA	4	8	16	16	16	16	16	16	16	16
T122	VISA	Slow VISA	4	8	8	8	8	6	6	8	8	8
T123	VISA	Slow VISA	4	6	8	8	8	6	8	8	8	8
T12-L	hVISA	hVISA	3	3	3	3	3	3	3	3	3	3
T15-L	hVISA	hVISA	3	3	3	3	4	4	4	4	4	4
T61-L	hVISA	hVISA	3	3	3	3	4	6	6	6	6	6
T89-L	hVISA	hVISA	3	3	3	3	3	3	3	3	3	3
T122-L	VSSA	VSSA	2	2	2	2	2	3	3	3	3	3
T123-L	VSSA	VSSA	2	2	2	2	2	3	3	3	3	3

a T-L strains are revertants obtained from the original clinical strains by subculture for 5 days in the absence of antibiotics.

b Slow-VISA strains showed increasing resistance to vancomycin after 72 h of incubation (vancomycin MICs of >4 μg/ml), although they had vancomycin MICs of >4 μg/ml after 48 h of incubation. The Mu3 and Mu50 isolates with the hVISA and VISA phenotypes had stable vancomycin MICs of 3 and 12 μg/ml, respectively, over time.

We confirmed the unstable expression of the slow-VISA phenotype in the absence of antibiotics. The levels of resistance to vancomycin in six slow-VISA strains (T series strains in Table 2) were reduced after a 5-day subculture in the absence of antibiotic. All six revertants (L strains derived from slow-VISA T strains) reverted to the VSSA or hVISA phenotype.

The zone of inhibition around a 30-μg/ml vancomycin disk in the disk diffusion test was hazy for slow-VISA Mu3-6R on a BHI agar plate (Fig. 5). The 30-μg/ml vancomycin disk diffusion test may be a useful method for detection of slow-VISA strains.

### Sequence analysis of emergence of the slow-VISA phenotype from VSSA via hVISA.

We previously reported that a single mutation of RpoB (R512P substitution) is responsible for nonsusceptibility to vancomycin (4). The Mu3-6R-L1 (hVISA) and Mu3-6R-L2 (hVISA) revertant mutants showed the large-colony phenotype and were isolated from slow-VISA strain Mu3-6R after antibiotic-free subculture for 5 days, as described previously (4). Mu3-6R-L2-6R (slow VISA) was obtained from a pinpoint colony of Mu3-6R-L2 growing on a BHI agar plate containing vancomycin at 6 μg/ml. We previously reported that when RpoB(R512P) in slow-VISA strain Mu3-6R was changed to an R512S, R512L, or R512H mutant, it resulted in the hVISA phenotype (4). We used deep sequencing to study the relationship between phenotype and the proportions of these various amino acid substitutions in the *rpoB* gene.

**FIG 6:**
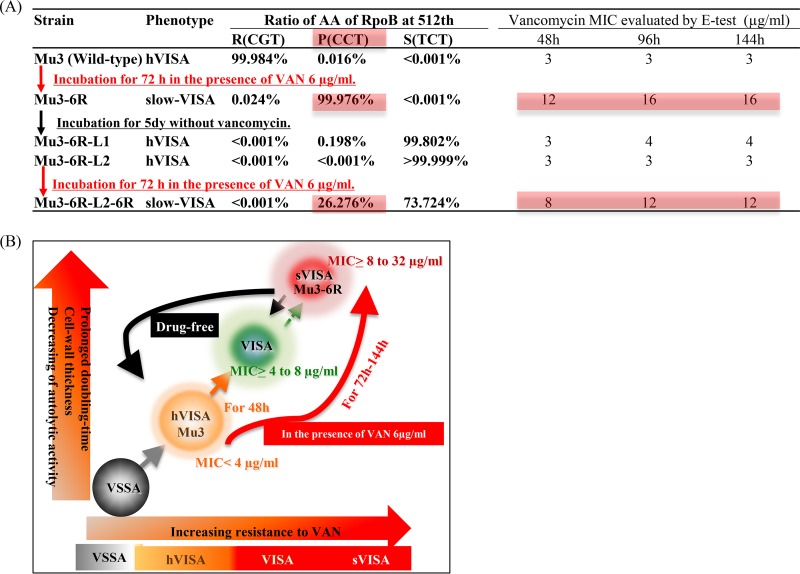
Emergence of the slow-VISA phenotype from VSSA via hVISA or VISA. (A) Proportions of mutations at the 512th residue of the RpoB gene, which confers high resistance to vancomycin, according to analysis of the *rpoB* amplicon by deep sequencing of Mu3-6R derivative clones. (B) The slow-VISA (sVISA) strain converted to VISA and hVISA in the absence of vancomycin.

At baseline, Mu3 or Mu3-6R grown in the presence of 6 μg/ml of vancomycin carried >99.9% RpoB(R512) or RpoB(R512P), respectively (Fig. 6A). The Mu3-6R-L1 (hVISA) revertant mutant cells had 0.20% RpoB(R512P) and 99.8% RpoB(R512S). The Mu3-6R-L2 (hVISA) revertant mutant had >99.9% RpoB(R512S), whereas Mu3-6R-L2-6R, expressing the slow-VISA phenotype, contained 26.3% RpoB(R512P) and 73.7% RpoB(R512S). Thus, various RpoB R512 substitution mutants were associated with the slow-VISA Mu3-6R phenotype (Fig. 6A). The slow-VISA phenotype emerged in the presence of vancomycin, and the phenotype was lost in the absence of vancomycin, reverting to the hVISA phenotype (Fig. 6B).

## DISCUSSION

We found that several mutations associated with the stringent response are found in slow-VISA mutant strains. A mupirocin-induced stringent response likely accounts for detection of the slow-VISA phenotype. The mechanism of resistance to vancomycin of slow-VISA strains differs from that of VISA strains because mupirocin induces the phenotype of the former but not the latter, and the associated mutations are different. For example, slow-VISA strain Mu3-6R has the RpoB(R512P) mutation, whereas VISA strain Mu3-5-5d has TagH(D26E).

Among the 23 slow-VISA isolates and their revertants, mutations in the *rpoB* and *rpoC* genes occurred more frequently in the slow-VISA strains than in the revertants, accounting for 32% of the total. Although *rpoB* is not a regulatory gene, its mutation drastically changes the transcription profile of the cell—much more than any mutations in regulators do. An *rpoB* mutation may act as a regulatory mutation (4, 5, 21, 22).

Besides RpoB mutation, we identified several other mutations in genes involved in various metabolic pathways, such as purine/pyrimidine synthesis in the pentose phosphate pathway. The increase in pools of CTP, UTP, and UMP shown in Table 1 is associated with the thickness of the cell wall and the VISA phenotype (5, 23–25). Pyrimidine upregulation has been reported to be normal with respect to the synthesis of ribosomal components but abnormal in terms of the ppGpp pool (26). Therefore, we speculate that the vancomycin resistance of slow VISA is due to an increase in pools of CTP, UTP, and UDP.

Mutations of *pfk* and *pykA* altered the amounts of the metabolites d-fructose-6P, d-fructose-1,6P_2_, and pyruvate in slow-VISA isolates. It is likely that d-fructose-6P pooled in the slow-VISA strain and contributed to cell wall synthesis.

The *capP* gene encodes an enzyme that converts UDP-GlcNac to UDP-GlcManNAc (27). Mutations in this gene may stimulate the pathway of cell wall synthesis at the expense of purine/pyrimidine synthesis by way of d-fructose-6P. Upregulated *glmS* increases production of glucosamine 6-P, which is required for the synthesis of peptidoglycan and wall teichoic acids, major components of the S. aureus cell wall (34). These mutations altering the metabolic pathways of slow VISA may result in activation of cell wall synthesis, causing thickening of the cell walls and consequent acquisition of resistance to vancomycin.

We compared the mutations in the 23 revertants (VSSA or hVISA strains) obtained from slow-VISA cells in the absence of antibiotics. These mutations localized in genes involved in the stringent response, likely affecting the flow of metabolites in the cell and promoting cell wall peptidoglycan synthesis by concentrating energy and metabolites within that pathway.

Colistin has also been reported to induce vancomycin tolerance that is associated with quorum sensing. Nevertheless, the mechanisms are different from mupirocin's mode of action (28). Further research into the mechanisms by which mupirocin induces the slow-VISA phenotype is needed.

We identified the following three characteristics of slow-VISA induction by mupirocin: (i) stable resistance to vancomycin, (ii) an accelerated growth rate, and (iii) tolerance of bactericidal activity. These were utilized to develop a method for detection of slow VISA in this study. Using this method, we found that the slow-VISA phenotype was prevalent in Japanese hospitals, being detected at a frequency of about 15.6% in the tested MRSA clinical isolates from blood cultures. We also isolated cells with the slow-VISA phenotype (vancomycin MIC of ≥16 μg/ml) from three VISA strains, namely, HIP07920 (isolated in 1999, in Rhode Island, from the blood of a patient with bacteremia), HIP12864 (isolated in 2003, in Oklahoma, from the blood of a patient with bacteremia) (29), and P1V44 (isolated in 1999, in Belgium, from sputum of a patient with cystic fibrosis) (30), in the absence of antibiotics (T. Azechi, unpublished data). Slow VISA may be prevalent in hospitals worldwide.

We also found that slow VISA emerged in the presence of vancomycin, lost resistance in the absence of vancomycin, and reverted to the hVISA or VSSA phenotype (Fig. 6). Slow-VISA cells were present in small numbers among the hVISA revertants, and their numbers increased again when they were incubated in the presence of vancomycin. Slow-VISA strains may account in part for recurrent and persistent MRSA infections (Fig. 6). Rapid detection of slow VISA may be an important tool for predicting and preventing vancomycin treatment failure.

## MATERIALS AND METHODS

### Bacterial strains and culture conditions.

Bacterial strains used in this study were routinely grown in BHI medium (Eiken Chemical Co., Ltd., Tochigi, Japan) at 37°C. Two clinical strain collections were tested for the presence of slow VISA. We selected 156 MRSA blood isolates (described as the T series) collected from 1987 to 2007, before the use of daptomycin and linezolid in Japan (see Table S1 in the supplemental material) (20), and we randomly selected 184 MRSA blood isolates from 803 clinical strains of MRSA collected from January 2008 through May 2011 at nine university hospitals in Japan (Table S2) (19). The study protocol was approved by the Institutional Review Board.

### DNA methods.

The standard methods for DNA manipulations were described previously (31). Genomic DNAs were prepared with a Miniamp kit (Qiagen Inc., Valencia, CA). Routine PCR amplification was conducted by means of the Expand High-Fidelity system (Roche, Mannheim, Germany).

### Antibiotic susceptibility tests.

MIC values were determined by Etest (AB Biodisk, Solna, Sweden). BHI agar was used instead of Mueller-Hinton agar because the former is more supportive of the expression of the VISA phenotype than are other agar types (32). Plates were then incubated at 37°C and analyzed after 48 h to 144 h. The Etest to determine the MIC of vancomycin (AB Biodisk, Solna, Sweden), the 30-μg/ml vancomycin agar disk diffusion susceptibility test (Eiken), and population analysis were carried out on BHI agar following the CLSI guidelines. Mupirocin (Roche) was used for the selection of slow VISA.

VSSA was defined as S. aureus with a vancomycin MIC of <4 μg/ml (CLSI breakpoint) that was unaffected by prolonged incubation. hVISA was defined as S. aureus with a vancomycin MIC of <4 μg/ml (CLSI breakpoint) that was unaffected by prolonged incubation or incubation with mupirocin but had a subpopulation of cells resistant to 4 μg/ml of vancomycin, at a frequency of 1 × 10^−7^ or greater. Slow VISA was defined as S. aureus with increasing resistance to vancomycin with prolonged incubation of >48 h, to an MIC of >8 μg/ml. Other properties of slow VISA are a low growth rate, unstable resistance and growth phenotypes, tolerance to killing by vancomycin, and a small or pinpoint colony morphology. VISA was defined as S. aureus with a stable vancomycin MIC of >4 μg/ml with prolonged incubation. Other properties are a stable phenotypic expression, colony morphology, and growth rate.

### Emergence of slow-VISA isolates from hVISA strain Mu3.

Twenty-three revertants were obtained from the 23 slow-VISA isolates, derived from Mu3 by selection with 6 μg/ml of vancomycin (4), by growth in the absence of antibiotics after subculture for 5 days. Doubling times and vancomycin MICs were determined (Table S3).

### Genome analysis.

We carried out single nucleotide polymorphism (SNP) detection according to a previously described protocol (4) and by deep sequencing of the amplified *rpoB* gene according to the manufacturer's recommendations (TaKaRa). In brief, 150-bp DNA fragments of the *rpoB* gene were amplified by PCR using the primers described previously (4). The amplicon library was constructed by means of a Nextera XT DNA sample preparation kit, and 150-bp paired-end read sequencing was performed on a Miseq sequencing platform (Illumina, Inc., San Diego, CA). Reads of 30 Mb were obtained from each amplicon DNA library. Each read was mapped to the reference *rpoB* sequence of strain N315.

### Metabolome analysis.

hVISA strain Mu3, VISA strain Mu3-3-5d (showing a stable vancomycin MIC of 8 μg/ml over time), and slow-VISA strain Mu3-6R carrying the RpoB(R512P) mutation each was grown as described above in defined synthetic medium. VISA strain Mu3-3-5d was isolated from hVISA Mu3 by selection on vancomycin at 6 μg/ml and was found to carry the TagH(D26E) mutation affecting the teichoic acid export ATP-binding protein (4). The cells were treated with 0.032 μg/ml mupirocin, and samples were harvested immediately after the addition of mupirocin (0 min) and after 30 min. The cells were washed and quenched, and metabolites were extracted according to the manufacturer's recommendations (Human Metabolome Technology [HMT], Tokyo, Japan). Briefly, the metabolite extraction procedure was conducted as follows. Cells were grown in 40 ml of medium in 100-ml Erlenmeyer flasks with shaking at 180 rpm and 37°C. For each strain, three cultures were incubated in parallel. Cells were harvested by vacuum filtration through a filter with a 0.45-μm pore size. The cells on the filter were immersed in 2 ml of methanol containing 10 μM internal standard solution 1 (HMT). Analytical biochemistry detection of nucleotides was performed by capillary electrophoresis with mass spectrometry (CE-MS; HMT). Identification and calibration of calibration curves for all the metabolites were carried out via comparison and measurement of a pure standard compound, (p)ppGpp (Trilink Biotechnology, San Diego CA). The results for intracellular metabolites were normalized to the cell dry weight (CDW). Experiments were performed in duplicate.

### Development of a method for detecting slow VISA by use of mupirocin.

Measurement of the appearance rate of hVISA (large-colony [LC] phenotype) from slow VISA (small colonies) and construction of growth curves were performed as described previously (4). Time-kill analyses were conducted to assess killing of bacteria (33). In brief, an inoculum of 10^6^ CFU/ml of fresh overnight culture was used. Four-milliliter aliquots of BHI broth were used for all the time-kill analyses. Bacterial colony counts were determined in duplicate at baseline and 12, 24, 48, and 72 h after incubation at 37°C. Mupirocin concentrations ranged from 0.016 to 0.064 μg/ml (Fig. S1). The vancomycin MIC was determined by Etest (AB Biodisk, Solna, Sweden), using BHI agar containing mupirocin at 0.032 μg/ml and drug-free BHI agar (Yuki Katayama, 7 March 2017, patent application PCT/JP2017/008975).

## Supplementary Material

Supplemental material
