# Supplementary material for: Prevalence of Slow-Growth Vancomycin Nonsusceptibility in Methicillin-Resistant Staphylococcus aureus
Source: Antimicrob Agents Chemother. 2017 Oct 24;61(11):e00452-17. doi: 10.1128/AAC.00452-17 (PMC5655046; doi:10.1128/AAC.00452-17)
Supplement: Supplemental material [file supp_61_11_e00452-17__index.html]

Supplemental material 

# Prevalence of Slow-Growth Vancomycin Nonsusceptibility in Methicillin-Resistant Staphylococcus aureus

## Supplemental material

- Supplemental file 1 -

  Supplemental Figure S1

  PDF, 190K
- Supplemental file 2 -

  Supplemental Tables S1 to S4

  XLSX, 83K
