# Supplementary material for: Prevalence of Slow-Growth Vancomycin Nonsusceptibility in Methicillin-Resistant Staphylococcus aureus
Source: Antimicrob Agents Chemother. 2017 Oct 24;61(11):e00452-17. doi: 10.1128/AAC.00452-17 (PMC5655046; doi:10.1128/AAC.00452-17)
Supplement: Supplemental material [file AAC.00452-17_zac011176622s1.pdf]

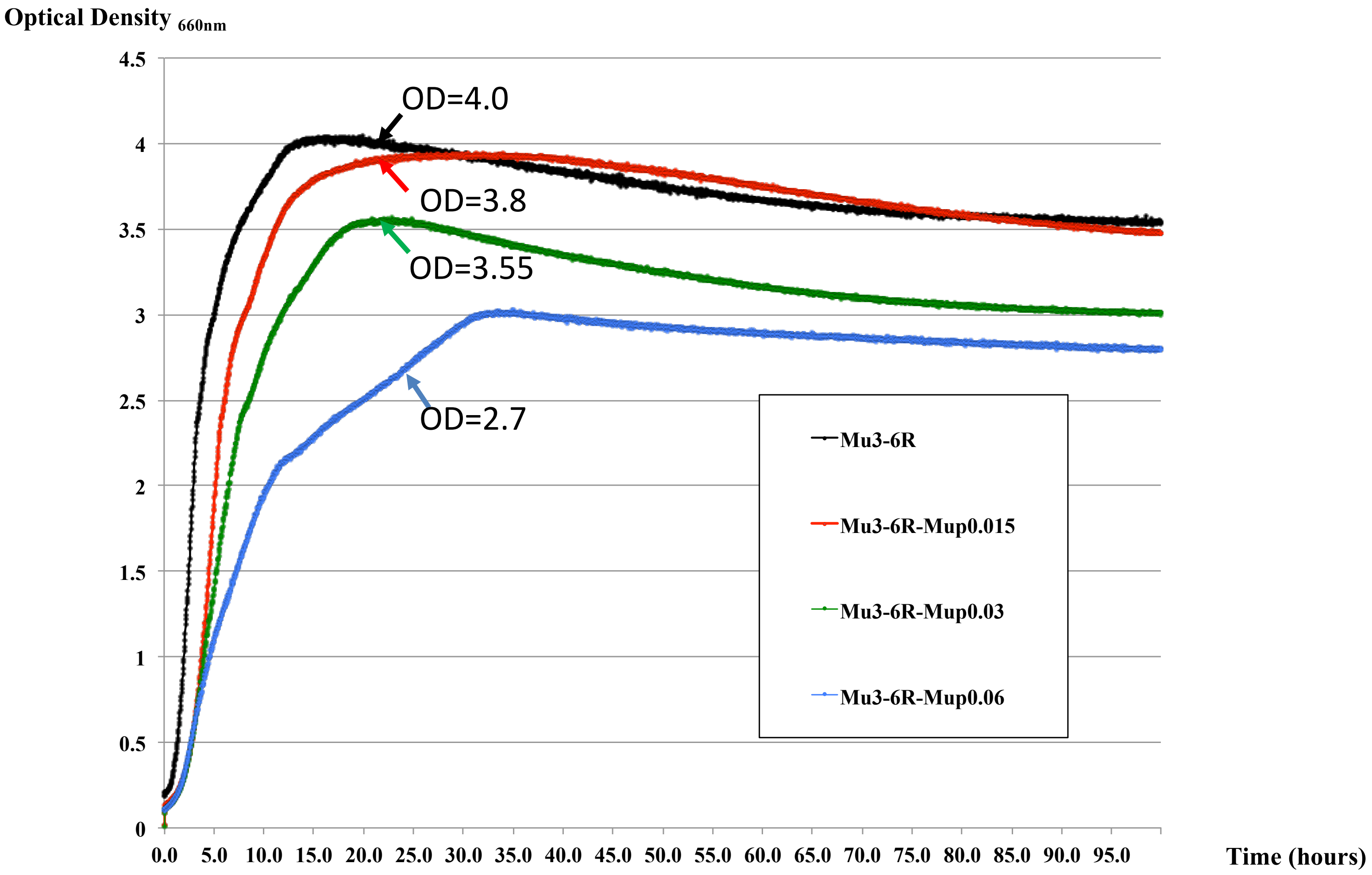

Figure S1.Effect of mupirocin on growth of slow-VISA Mu3-6R.

Mu3-6R was inoculated in 10-ml of BHI broth black indicates drug-free; red, mupirocin 0.015μg/ml; green, mupirocin 0.03μg/ml; blue, including 0.06μg/ml. OD was indicated at 25 hours.
